# Myasthenia gravis and anxiety-depression states: an integrated clinical and Mendelian randomization study

**DOI:** 10.3389/fneur.2026.1791340

**Published:** 2026-03-18

**Authors:** Lijun Luo, Xinyi Zhu, Wenfeng Chen, Tong Ye, Jie Yang, Dongsheng Wei, Yaxin Zeng, Ping Yu

**Affiliations:** 1 Department of Neurology, Wuhan No. 1 Hospital, Wuhan, China; 2 Department of Neurology, Wuhan Jinyintan Hospital, Tongji Medical College of Huazhong University of Science and Technology, Wuhan, China; 3 The First Clinical Medical Institute, Hubei University of Chinese Medicine, Wuhan, China

**Keywords:** anxiety disorder, depression, genome-wide association studies, Mendelian randomization, myasthenia gravis

## Abstract

**Background:**

Myasthenia gravis (MG) is an autoimmune disease affecting the postsynaptic membrane at the neuromuscular junction. Anxiety disorder and depression are common psychiatric comorbidities in patients with MG. However, the potential causal relationship between MG and these mental health conditions remains unclear.

**Methods:**

A retrospective analysis was first conducted to collect relevant data from 96 patients with MG at the time of their initial definitive diagnosis at our center. The cohort was divided into an anxiety group and a non-anxiety group, and into a depression group and a non-depression group. Statistical methods were employed to examine the relationship between MG scale scores and the presence of anxiety and depression. Subsequently, for the first time, we employed a bidirectional two-sample Mendelian Randomization (MR) approach to explore the causal relationships between MG, anxiety disorders, and depression. We utilized the largest and most recent European population genome-wide association studies available for the three phenotypes to extract genetic variants that could serve as instrumental variables. The inverse variance weighted model was used as the primary analysis method. Multiple sensitivity analyses and pleiotropy tests were conducted to validate the robustness of the primary findings.

**Results:**

In clinical studies, it was observed that 62.5% of MG patients exhibited anxiety symptoms, while 46.90% presented depressive symptoms with myasthenia gravis (MG). Through univariate regression analysis, no direct associations were identified between gender, age, disease duration, or MGFA classification and the occurrence of anxiety and depression. However, Quantitative Myasthenia Gravis (QMG) Score and Myasthenia Gravis Activities of Daily Living (MG-ADL) score were significantly associated with anxiety and depression among patients with MG (*p* < 0.05). After adjusting for confounding variables, each one-point increase in the QMG score was associated with a 11.9% higher risk of anxiety and a 15.7% higher risk of depression, and each one-point increase in the MG-ADL score, the risk of anxiety and depression increases by 22.5 and 18.5%. These findings suggest that more severe MG symptoms and reduced quality of life are more likely to be accompanied by anxiety and depression. Further Mendelian randomization (MR) analyses provided no evidence supporting the hypothesis that genetically predicted MG influences the risk of anxiety disorders or depression. Reverse MR analysis also failed to reveal any causal relationship between these psychiatric comorbidities and MG. Sensitivity and pleiotropy analyses confirmed the robustness of our results.

**Conclusion:**

In real-world clinical research, patients with MG who have more severe disease are more likely to present with anxiety and depression, and the two conditions show a clear association. However, from a genetic perspective, Mendelian Randomization (MR) analysis does not support a causal relationship between MG and anxiety or depression in European populations. The correlation observed in our clinical research may reflect non-causal associations not explained by genetic liability, potentially involving shared genetic architecture or non-genetic mechanisms.

## 1 Introduction

Myasthenia gravis (MG) is a chronic autoimmune disease characterized by the impairment of neuromuscular transmission. This impairment is caused by antibodies that target components of the neuromuscular junction, postsynaptic membrane, including the acetylcholine receptor (AchR), muscle-specific kinase (MuSK) and low-density lipoprotein receptor-related protein 4 (LRP4). MG is typically characterized by fatigability and fluctuating weakness of skeletal muscles, including the ocular, bulbar, and limb muscles (1). Notably, when respiratory muscles are involved, the result can be potentially life-threatening acute respiratory failure. According to global epidemiological studies, the mean prevalence of MG is 173.3 cases per million population, and the mean mortality rate is 1.4 deaths per million population per year (2). Patients with MG often present with psychiatric comorbidities, with anxiety disorders and depression being the most common (3).

Patients with MG have a relatively high prevalence of both anxiety and depression (4). In a global, multicenter, prospective observational study, the Berri-Aknin team found that of 889 MG patients assessed using the anxiety and depression scale, 52.7% were diagnosed with anxiety, while 43.2% presented with depression (3). Another study found that the prevalence of moderate to severe depression in MG patients is approximately 20.5% (5). Furthermore, a significant correlation has been observed between depression, anxiety symptoms (6), and MG severity, with depression scores serving as a reliable predictor of disease severity (7). The comorbidity between MG and psychiatric disorders warrants close attention. However, the causal relationship between MG and the development of anxiety and depression remains to be elucidated.

For rare diseases such as MG, conducting randomized controlled trials is often impractical. Mendelian randomization (MR) provides a cost-effective and convenient method to assess causal relationships between exposures and outcomes. By using genetic variants as instrumental variables (IVs), MR addresses potential confounding factors and reverse causation, providing robust evidence for causal inference (8). In this study, we used two-sample Mendelian randomization to examine the bidirectional causal relationship between MG and mental disorders, specifically anxiety and depression.

## 2 Materials and methods

### 2.1 Study design

Patients with MG admitted to the Department of Neurology at Wuhan No. 1 Hospital between August 2021 and August 2024 were enrolled (*n* = 96). Baseline data were collected, and patients were categorized into four groups. Statistical analyses were used to assess correlations between MG-related scale scores and anxiety and depression and to identify risk factors for anxiety and depression in MG. Subsequently, a bidirectional two-sample Mendelian randomization (MR) analysis was conducted using genome-wide association study (GWAS) data to test the causal relationships between MG and anxiety and depression.

The inclusion criteria were as follows: Patients diagnosed MG who were also: (1) aged 18–85 years, with an MGFA classification of IIa-IVb; (2) able to understand and sign informed consent forms. Exclusion criteria were as follows: (1) concurrent malignancy (except thymoma); (2) those with a previous diagnosis of anxiety/depression prior to the diagnosis of MG; (3) severe active hepatitis B or C, active tuberculosi; (4) severe liver or kidney dysfunction, multiple organ failure; (5) severe allergy or infection; (6) pregnant and lactating women.

Patient demographics (age, gender), disease duration, MGFA classification, thymus CT scans, corticosteroid use, QMG score, MG-ADL score, and Hamilton Depression Scale 24-item (HAMD) (9), Hamilton Anxiety Scale 14-item (HAMA) (10) scores for all patients were collected. The Hamilton Depression Rating Scale (HAMD) is a clinician-administered instrument commonly used to assess depressive symptomatology. It comprises 24 items and yields a maximum total score of 86. Higher scores indicate more severe depressive symptoms. The Hamilton Anxiety Rating Scale (HAMA) consists of 14 items, each rated on a five-point scale (0–4), yielding a total score of 0–56. Higher scores reflect greater anxiety severity, with scores of 0–7 indicating the absence of clinically significant anxiety. Based on the HAMA and HAMD scores, participants with HAMA ≥14 were classified as the anxiety group and those with HAMA <14 as the non-anxiety group; participants with HAMD ≥20 were classified as the depression group and those with HAMD <20 as the non-depression group.

#### 2.1.1 Statistical analysis of retrospective studies

Statistical analyses were performed using IBM SPSS Statistics (version 25). Tests of normality were conducted for all continuous variables. Normality tests were conducted for all continuous variables. Variables conforming to a normal distribution were expressed as means ± standard deviations (SD), and those not conforming were expressed using medians and interquartile ranges (IQR). Percentages were used when necessary. For between-group comparisons, the independent-samples *t* test was used for normally distributed data, the Mann–Whitney U test for non-normally distributed data, and the χ^2^ test for categorical variables. Logistic regression was employed to investigate clinical factors. Variables with *p*-values <0.1 in univariate analyses, along with clinically relevant confounders, were included in the multivariable logistic regression model. Results are reported as odds ratios (OR) with 95% confidence intervals (CI). *p* <0.05 were considered statistically significant.

### 2.2 Statistical analysis of Mendelian randomization studies

**Figure 1:**
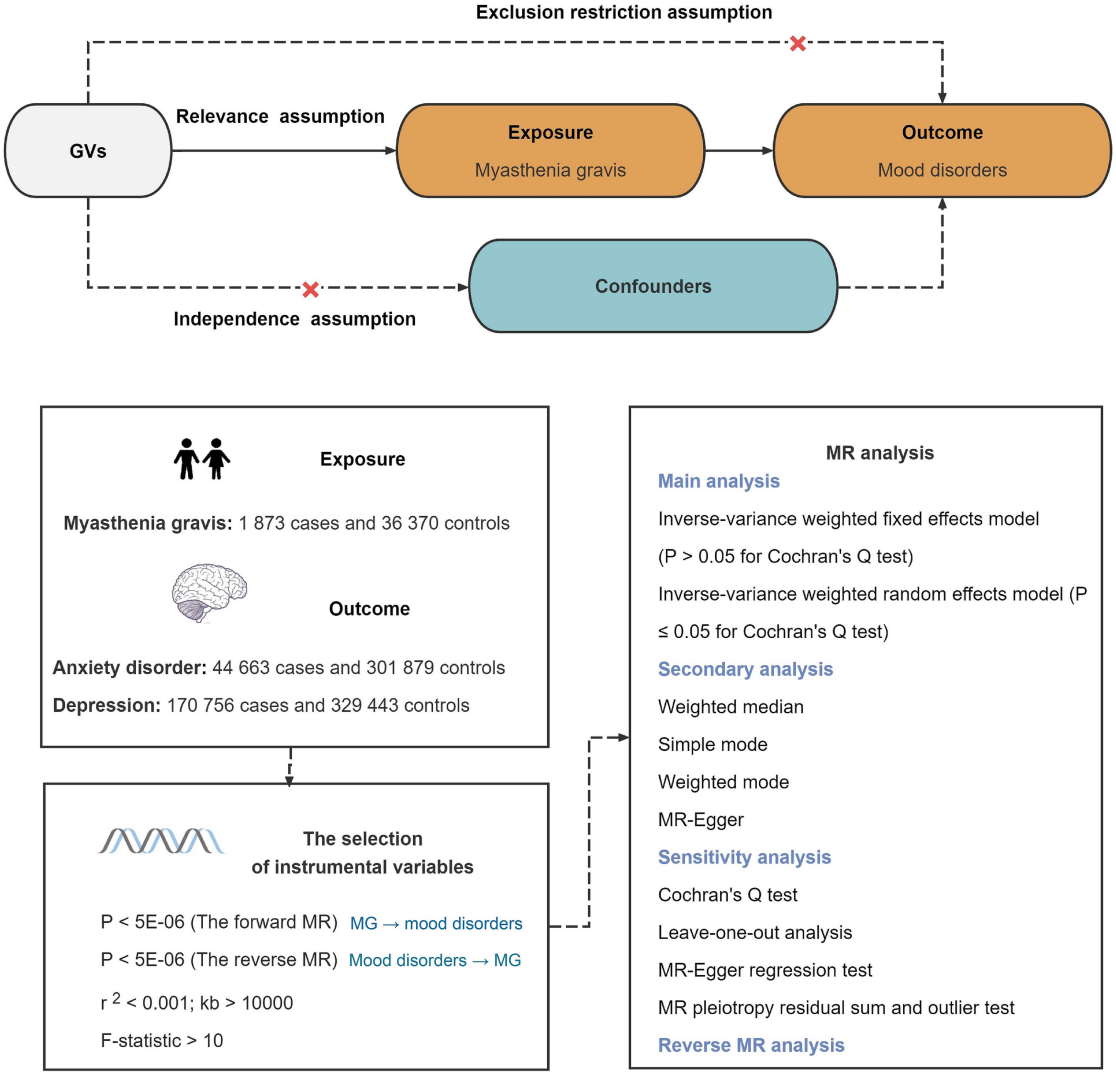
Schematic of this Mendelian randomization study. GVs, genetic variants.

Figure 1 outlines the basic principle and overall design process of this study. Forward MR analysis relies on three basic principles: (1) the IVs must have a strong association with MG; (2) the IVs should remain unrelated to the confounding factors; (3) the IVs should not influence anxiety and depression outside the effects of MG. The same applies to reverse MR. This research strictly adheres to the STROBE MR checklist in reporting (Supplementary Table S1) (11).

#### 2.2.1 Data sources

The detailed information along with the sources for downloading the genome-wide association studies (GWAS) dataset are documented in Table 1. For MG, we utilized the largest available GWAS cohort comprising 1,873 patients with MG and 36,370 healthy individuals, covering approximately 24 million single nucleotide polymorphisms (SNPs) (12). The data originate from discovery cohorts in the USA and Italy, with results verified in a replication cohort from the UK. Patients with systemic MG are diagnosed with fluctuating muscle weakness, electrophysiological and/or pharmacological abnormalities. AChR antibodies are detected in approximately 90% of patients.

**Table 1:** The detailed information of the genome-wide association study used in this MR study.

Category	Ethnicity	Sample size	URL (data download)	Pubmed ID
Exposure				
Myasthenia gravis	European	1,873 cases and 36,370 controls	https://gwas.mrcieu.ac.uk/datasets/ebi-a-GCST90093061/	35,074,870
Outcome				
Anxiety disorder	European	44,663 cases and 301,879 controls	https://r10.finngen.fi/	36,653,562
Depression	European	170,756 cases and 329,443 controls	https://gwas.mrcieu.ac.uk/datasets/ieu-b-102/	30,718,901

For anxiety disorders, the GWAS data were derived from FinnGen, which includes 44,663 cases and 301,879 controls. Anxiety disorders are defined as a category of psychiatric disorders characterized by feelings of fear or anxiety, often accompanied by physical symptoms associated with anxiety.

For depression, the GWAS datasets were derived from the UK Biobank and the Psychiatric Genomics Consortium (13). This included an analysis of 170,756 cases and 329,443 controls. The UK Biobank diagnosed depression based on self-reported symptoms, treatments, or hospital records (14). The Psychiatric Genomics Consortium, however, used a structured diagnostic interview approach (15).

#### 2.2.2 Selection of instruments

In the forward MR analysis, referencing the previous MR approach, SNPs meeting a genome-wide significance threshold of *p* < 5E-06 were selected from the MG datasets to increase the number of SNPs available for sensitivity analysis (16). The European reference panel of the 1,000 Genomes Project was used (R2 < 0.001, window = 10,000 kb). We excluded SNPs with minor allele frequency <0.01. In addition, we removed palindromic SNPs to align the MG data with the outcomes. The PhenoScannerV2 database was used to exclude SNPs with significant associations with confounding variables or outcomes (17). The F-statistic was used to assess the strength of IVs, excluding SNPs with an F-statistic <10 (indicating weak IVs) (18). In reverse MR, we screened for IVs in anxiety disorder and depression using the genome-wide significance threshold of *p* < 5E-08, with the remaining steps identical to those in forward MR analysis.

#### 2.2.3 Statistical analysis

In the forward MR analysis, we used the Inverse-Variance Weighted (IVW) method to assess the causal relationship between MG and outcomes (anxiety disorder and depression). The Cochran *Q*-test *p*-value was used to examine the heterogeneity of the results. If the Cochran *Q*-test *p* > 0.05, there is no heterogeneity in the results and we used the IVW Fixed Effects model (IVW-FE) to calculate the effect size. However, if the Cochran *Q*-test *p* ≤ 0.05, indicating heterogeneity in the results, the IVW Random Effects model (IVW-RE) was used (19).

A series of sensitivity analyses were performed to validate the robustness of the IVW results, including MR-Egger, weighted median, simple mode, and weighted mode (20). The MR-Egger regression (21) and the MR-PRESSO Global test (22) were used to examine whether pleiotropy affected the results. The Leave-One-Out (LOO) test was used to check whether a single SNP significantly drove the causal relationship.

The odds ratio (OR) and 95% confidence interval (CI) were used to quantify risk. The statistical significance was defined as *p* < 0.05. MR analyses were performed in R (version 4.3.1) using the TwoSampleMR package.

#### 2.2.4 Selection of instrumental variables

In our MR analyses, the number of SNPs used as IVs ranged from 8 to 35, and the F-statistics of all IVs were greater than 10 (ranging from 173.72 to 1349.13) (Table 2). These statistics indicated that there was sufficient power in the use of these IVs. Detailed information on all SNPs included in the MR analyses can be found in Supplementary Table S2.

**Table 2:** Causal effects between myasthenia gravis and mood disorders (anxiety disorder and depression).

Exposure	Outcome	F-statistic (nSNP)	R2	Methods	OR (95%CI)	*P*-value
Forward MR						
Myasthenia gravis	Anxiety disorder	1349.13 (26)	0.48	MR Egger	0.988 (0.952, 1.025)	0.536
				Weighted median	1.006 (0.984, 1.028)	0.597
				IVW-FE	1.005 (0.991, 1.019)	0.502
				Simple mode	1.003 (0.956, 1.053)	0.902
				Weighted mode	0.995 (0.964, 1.027)	0.766
	*Remove rs76815088*	1018.33 (25)	0.40	MR Egger	1.022 (0.967, 1.081)	0.438
				Weighted median	1.012 (0.988, 1.038)	0.331
				IVW-FE	1.013 (0.997, 1.029)	0.123
				Simple mode	1.010 (0.968, 1.053)	0.662
				Weighted mode	1.015 (0.977, 1.055)	0.456
Myasthenia gravis	Depression	1286.98 (24)	0.45	MR Egger	0.991 (0.952, 1.031)	0.664
				Weighted median	0.996 (0.982, 1.010)	0.566
				IVW-RE	0.998 (0.983, 1.014)	0.805
				Simple mode	0.989 (0.964, 1.014)	0.391
				Weighted mode	0.994 (0.975, 1.012)	0.508
	*Remove rs2523595*	1181.87 (23)	0.42	MR Egger	0.993 (0.960, 1.027)	0.686
				Weighted median	0.997 (0.983, 1.011)	0.696
				IVW-RE	1.004 (0.990, 1.017)	0.592
				Simple mode	0.988 (0.962, 1.016)	0.408
				Weighted mode	0.994 (0.975, 1.013)	0.517
Reverse MR						
Anxiety	Myasthenia gravis	282.54 (8)	0.01	MR Egger	10.119 (0.453, 226.253)	0.195
				Weighted median	1.128 (0.477, 2.669)	0.785
				IVW-FE	1.183 (0.555, 2.525)	0.663
				Simple mode	0.466 (0.095, 2.285)	0.378
				Weighted mode	0.623 (0.156, 2.488)	0.525
Depression	Myasthenia gravis	173.72 (35)	0.01	MR Egger	0.014 (0.001, 0.46)	0.022
				Weighted median	0.977 (0.469, 2.034)	0.951
				IVW-RE	0.839 (0.428, 1.643)	0.608
				Simple mode	0.969 (0.183, 5.119)	0.970
				Weighted mode	0.895 (0.167, 4.803)	0.898

nSNP, number of SNP; OR, odds ratio; CI, confidence interval; Inverse Variance Weighted Fixed Effects, IVW-FE; Inverse Variance Weighted Random Effects, IVW-RE.

### 2.3 Ethical approval

In this study, all participants provided written informed consent before enrollment. The study protocol was approved by the local institutional ethics committee (Ethics Committee of Wuhan No. 1 Hospital, approval no. 2021–34; approved on September 9, 2021). For the Mendelian randomization (MR) analyses, all data were obtained from publicly available sources; therefore, no additional ethical approval was required.

## 3 Results

### 3.1 Clinical study results

#### 3.1.1 Baseline characteristics of patients

The study included 96 patients with generalized myasthenia gravis (gMG). The median age was 57 years (interquartile range: 44–68); 43.80% of our patients (42 patients) were male, and 56.30% (54 patients) were female. The median disease duration was 4 years (interquartile range: 3–8.75). According to MGFA classifications, 49.00% of patients (47 patients) were class II, 47.90% (46 patients) were class III, 3.10% (3 patients) were class IV. Thymic abnormalities were present in 32.30% (31 patients) of the cases. Corticosteroids were used in 42.70% (41 patients). The mean score on the MG-ADL scale was 11.50 ± 3.22, and the mean score on the QMG scale was 17.55 ± 5.19. Anxiety symptoms were present in 62.5% (60 patients), Depressive symptoms were present in 46.9% (45 patients). The QMG and MG-ADL scores were significantly higher in the anxiety group than in the non-anxiety group. Similarly, the QMG and MG-ADL scores were significantly higher in the depression group than in the non-depression group (*p* < 0.05). In contrast, no statistically significant differences were observed in gender, age, disease duration, thymic abnormalities, corticosteroid use, or MGFA classification, indicating that these baseline characteristics were generally balanced (Table 3).

**Table 3:** Comparison of baseline characteristics stratified by anxiety and depression status in patients with MG.

Characteristic	Non-anxiety group (*n* = 36)	Anxiety group (*n* = 60)	χ^2^/t/Z value	*p*-value	Non-depression group (*n* = 51)	Depression group (*n* = 45)	χ^2^/t/*Z*-value	*P*-value
Sex			0.553	0.457			0.293	0.588
Male	(*n* = 14)	(*n* = 28)			(*n* = 21)	(*n* = 21)		
Female	(*n* = 22)	(*n* = 32)			(*n* = 30)	(*n* = 24)		
Age	58(33.25–69)	56.50(45.75–67.50)	−0.413	0.680	56(35–66)	59(49–69.50)	−0.981	0.327
Disease duration	4.5(3–6.75)	4(3–9)	−0.408	0.684	4.5(3–8)	4(3–9.50)	−0.477	0.643
Thymic abnormalities			0.079	0.778			0.042	0.838
Yes	(*n* = 11)	(*n* = 20)			(*n* = 16)	(*n* = 15)		
No	(*n* = 25)	(*n* = 40)			(*n* = 35)	(*n* = 30)		
corticosteroid use			0.071	0.790			0.254	0.614
Yes	(*n* = 16)	(*n* = 25)			(*n* = 23)	(*n* = 27)		
No	(*n* = 20)	(*n* = 35)			(*n* = 28)	(*n* = 18)		
MGFA classifications			2.036	0.261			2.892	0.236
Class II	(*n* = 21)	(*n* = 26)			(*n* = 29)	(*n* = 18)		
Class III	(*n* = 14)	(*n* = 32)			(*n* = 21)	(*n* = 25)		
Class IV	(*n* = 1)	(*n* = 2)			(*n* = 1)	(*n* = 2)		
QMG score	15.67 ± 4.81	18.68 ± 5.12	−2.859	0.005	15.84 ± 4.718	19.49 ± 5.066	−3.650	0.001
MG-ADL score	10.19 ± 3.031	12.05 ± 2.919	−2.944	0.004	10.59 ± 3.014	12.22 ± 2.953	−2.679	0.009

Values are presented as mean ± SD for normally distributed continuous variables, median (IQR) for non-normally distributed continuous variables. Anxiety was defined as HAMA ≥14 and non-anxiety as HAMA <14; depression was defined as HAMD ≥20 and non-depression as HAMD <20. QMG, Quantitative Myasthenia Gravis; MG-ADL, Myasthenia Gravis Activities of Daily Living; MGFA, Myasthenia Gravis Foundation of America clinical classification.

#### 3.1.2 Univariate logistic regression analysis of anxiety and depression in MG patients

Univariate logistic regression analyses were conducted using the presence of anxiety and depression as the dependent variable (anxiety or depression = 1, absence = 0), with general characteristics [gender, age, MGFA classification, disease duration, with or without thymic abnormalities, Corticosteroid use (yes/no)] and MG-ADL and QMG scores as independent variables. The findings of the current study indicated that the presence of anxiety or depression was positively associated with QMG and MG-ADL scores (*p* < 0.05). The investigation revealed no statistically significant correlation or causal relationship between general characteristics and the occurrence of anxiety or depression (*p* > 0.05) (Supplementary Table S3).

#### 3.1.3 Multivariate logistic regression analysis of factors associated with anxiety and depression in patients with MG

To identify independent predictors of anxiety and depression, four multivariable logistic regression models were constructed, and key confounders were adjusted for based on univariable screening (*p* < 0.10) and prior literature. The analysis indicated that each 1-point increase in the QMG score was associated with a 11.9% (*p* = 0.024) higher odds of anxiety and a 15.7% (*p* = 0.005) higher odds of depression; each 1-point increase in the MG-ADL score was associated with a 22.5% (*p* = 0.014) higher odds of anxiety and a 18.5% (*p* = 0.031) higher odds of depression, These findings suggest that patients with higher QMG or MG-ADL scores have a greater likelihood of anxiety and depression (Table 4). In addition, to address the potential dose–response relationship, we additionally performed linear regression analyses using HAMA and HAMD total scores as continuous outcomes, with QMG and MG-ADL as predictors. These analyses showed that each 1-point increase in QMG score was associated with a 0.703-point increase in the HAMA score (*p* = 0.002) and a 0.622-point increase in the HAMD score (*p* = 0.010). Similarly, each 1-point increase in MG-ADL score was associated with a 1.079-point increase in the HAMA score (*p* = 0.003) and a 0.812-point increase in the HAMD score (*p* = 0.035). These findings indicate that higher QMG and MG-ADL scores were associated with higher HAMA and HAMD scores (Table 5).

**Table 4:** Multivariate logistic regression analysis of factors associated with anxiety and depression in patients with MG.

Variable	Anxiety			Depression		
	B (coefficient)	OR (95% CI)	*P*-value	B (coefficient)	OR (95% CI)	*P*-value
QMG model						
QMG score	0.113	1.119(1.015–1.235)	0.024	0.146	1.157(1.046–1.280)	0.005
Age	0.005	1.005(0.976–1.034)	0.755	0.011	1.011(0.982–1.042)	0.451
Sex	0.330	1.390(0.568–3.402)	0.470	0.234	1.263(0.517–3.087)	0.608
Disease duration	0.005	1.005(0.948–1.065)	0.876	0.006	1.006(0.951–1.063)	0.840
MGFA classifications	0.139	1.149(0.432–3.053)	0.781	0.152	1.164(0.448–3.024)	0.755
With or without thymic abnormalities	0.036	1.036(0.402–2.674)	0.941	0.005	1.005(0.393–2.565)	0.625
Corticosteroid use (yes/no)	−0.108	0.898(0.358–2.252)	0.818	−0.231	0.794(0.314–2.003)	0.625
ADL model						
MG-ADL scores	0.203	1.225(1.043–1.440)	0.014	0.170	1.185(1.015–1.384)	0.031
Age	0.009	1.009(0.980–1.038)	0.546	0.017	1.017(0.989–1.046)	0.247
Sex	0.266	1.304(0.530–3.207)	0.563	0.165	1.179(0.494–2.813)	0.710
Disease duration	0.008	1.008(0.950–1.069)	0.799	0.006	1.006(0.952–1.063)	0.833
MGFA classifications	0.261	1.298(0.512–3.290)	0.583	0.434	1.543(0.630–3.782)	0.343
With or without thymic abnormalities	0.009	1.009(0.385–2.646)	0.985	0.017	1.017(0.404–2.561)	0.972
Corticosteroid use (yes/no)	−0.183	0.833(0.331–2.096)	0.698	−0.279	0.757(0.309–1.853)	0.542

QMG, Quantitative Myasthenia Gravis; MG-ADL, Myasthenia Gravis Activities of Daily Living; OR, odds ratio; CI, confidence interval. Binary outcomes were coded as follows: anxiety (1 = anxiety, 0 = non-anxiety; defined as HAMA ≥14) and depression (1 = depression, 0 = non-depression; defined as HAMD ≥20). Multivariable models were adjusted for age, sex, disease duration, Use of corticosteroid hormone therapy, with or without thymic abnormalities, and MGFA classifications.

**Table 5:** Linear regression analyses (continuous outcomes: HAMA/HAMD).

Outcome	Predictor	Model	B (95% CI)	β	*P*	Model fit
HAMA score	QMG score	Model 1 (unadjusted)	0.710 (0.332–1.089)	0.359	0.01	*R*^2^ = 0.129; *F*(1, 94) = 13.872; *P* < 0.001
		Model 2 (adjusted)	0.703 (0.264–1.141)	0.355	0.002	*R*^2^ = 0.145; Adj *R*^2^ = 0.077; *F*(7, 88) = 2.135; *p* = 0.048
HAMD score	QMG score	Model 1 (unadjusted)	0.699 (0.297–1.102)	0.335	0.001	*R*^2^ = 0.112; *F*(1, 94) = 11.891; *p* = 0.001
		Model 2 (adjusted)	0.622 (0.155–1.088)	0.298	0.010	*R*^2^ = 0.129; Adj R^2^ = 0.060; F(7,88) = 1.869; *p* = 0.084;
HAMA score	MG-ADL score	Model 1 (unadjusted)	1.111 (0.467–1.755)	0.333	0.001	*R*^2^ = 0.111; *F*(1, 94) = 11.728; *P* = 0.001
		Model 2 (adjusted)	1.079 (0.376–1.782)	0.323	0.003	*R*^2^ = 0.138; Adj *R*^2^ = 0.069; *F*(7, 88) = 2.007; *p* = 0.063
HAMD score	MG-ADL score	Model 1 (unadjusted)	0.951 (0.258–1.644)	0.271	0.008	*R*^2^ = 0.073; *F*(1, 94) = 7.425; *p* = 0.008
		Model 2 (adjusted)	0.812 (0.057–1.566)	0.231	0.035	*R*^2^ = 0.106; Adj R^2^ = 0.035; *F*(7, 88) = 1.497; *p* = 0.179

Model 2 adjusted for sex, age, disease duration, MGFA classifications, Use of corticosteroid hormone therapy, and with or without thymic abnormalities. VIF values were low (≈1.02–1.29), suggesting no multicollinearity.

### 3.2 Mendelian randomization analysis of anxiety or depression in MG patients

#### 3.2.1 Causal effects of MG on anxiety disorder and depression

**Figure 2:**
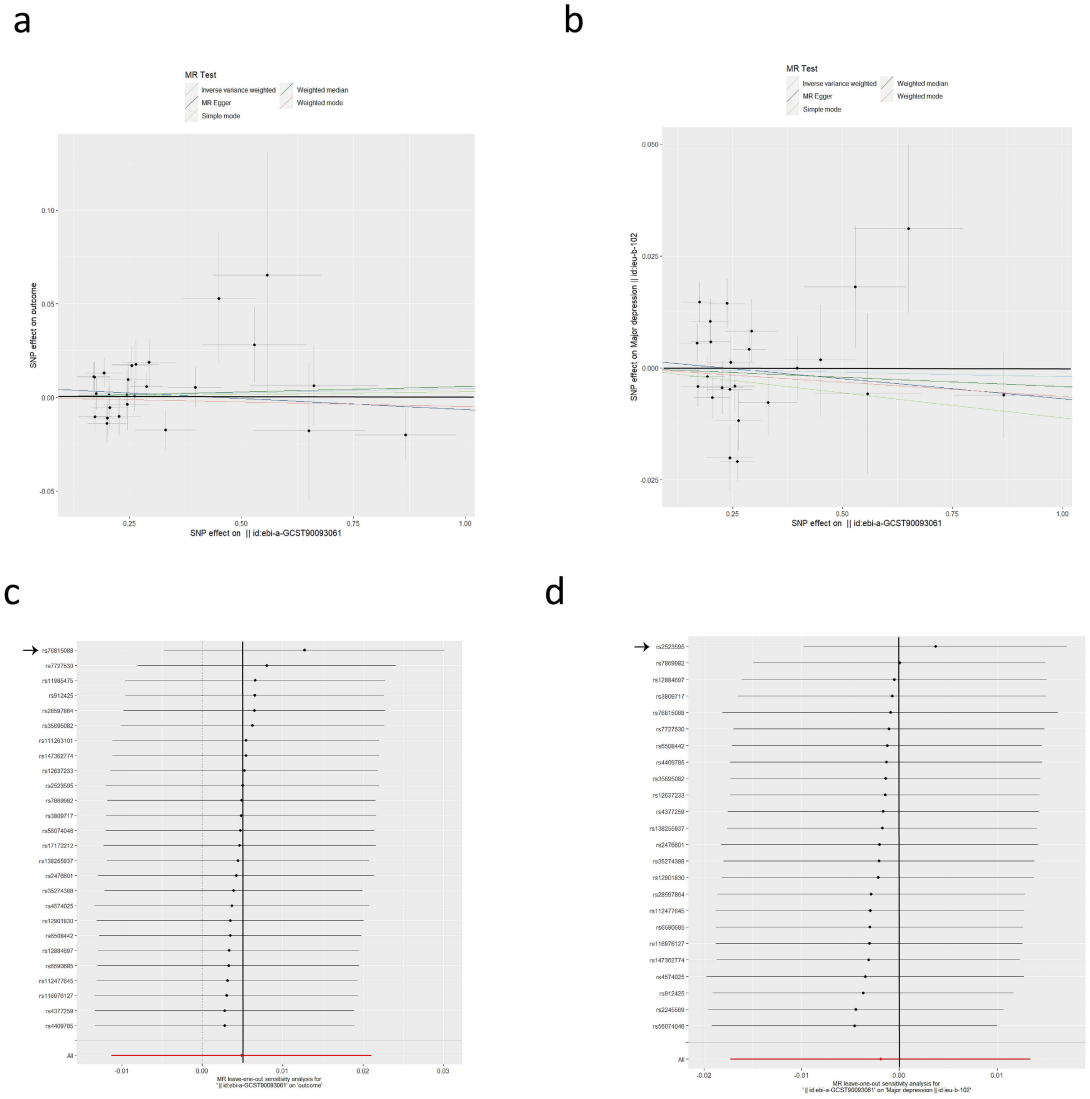
Causal effects of MG on anxiety disorder and depression. **(a)** Scatter plot of the causal relationship between MG and anxiety disorder using five MR methods. **(b)** Scatter plot of the causal relationship between MG and depression using five MR methods. **(c)** Leave-one-out plot of the causal relationship between MG and anxiety disorder. One SNP significantly drove the results (rs76815088). **(d)** Leave-one-out plot of the causal relationship between MG and depression. One SNP significantly drove the results (rs2523595).

Considering MG as an exposure to investigate its causal effects on anxiety and depression, we separately screened 26 and 24 SNPs, respectively. The causal effects between MG and anxiety disorder showed no heterogeneity (Cochran *Q*-test *p* = 0.151), so the IVW-FE (OR = 1.005, 95%CI: 0.991–1.019, *p* = 0.502) were used as our primary analysis (Figure 2a). The causal effects between MG and depression show heterogeneity (Cochran *Q*-test *p* < 0.001), so the IVW-RE (OR = 0.998, 95%CI: 0.983–1.014, *p* = 0.805) was used (Figure 2b). These results suggest that there is no apparent causal relationship between MG and both anxiety disorder and depression. The remaining sensitivity analyses were consistent with the primary analysis, confirming the robustness of the results (Table 2).

In the detection of horizontal pleiotropy, MR-Egger regression analyses revealed no horizontal pleiotropy (*p* > 0.05), and no outliers were observed in the MR-PRESSO analysis (Supplementary Table S4). The LOO analysis for the effect of MG on anxiety revealed that one SNP significantly drove the results (rs76815088) (Figure 2c). Therefore, after excluding this SNP, we performed the MR analysis again, and the result of IVW-FE (OR = 1.013, 95% CI: 0.997–1.029, *p* = 0.123) still indicated that MG had no effect on the risk of anxiety disorder. In the LOO analysis for the effect of MG on depression, one SNP was found to be significantly driving the results (rs2523595) (Figure 2d). After excluding this SNP, we performed the MR analysis again, and the IVW-RE result (OR = 1.004, 95% CI: 0.990–1.017, *p* = 0.592) indicated that MG had no effect on the risk of depression.

#### 3.2.2 Causal effects of anxiety disorder and depression on MG

Considering anxiety disorder and depression as exposures to investigate their causal effects on MG, we screened out 8 and 35 SNPs, respectively. The causal effects between anxiety disorder and MG did not show heterogeneity (Cochran *Q*-test *p* = 0.196), so we used the IVW-FE model (OR = 1.183, 95%CI: 0.555–2.525, *p* = 0.663) as our main analysis. The causal effects between depression and MG showed heterogeneity (Cochran *Q*-test *p* < 0.001), so the IVW-RE (OR = 0.839, 95%CI: 0.428–1.643, *p* = 0.608) was adopted. These analyses suggest that anxiety disorder and depression do not affect the risk of MG. The remaining sensitivity analyses were consistent with the main analysis, confirming the robustness of the results (Table 2).

**Figure 3:**
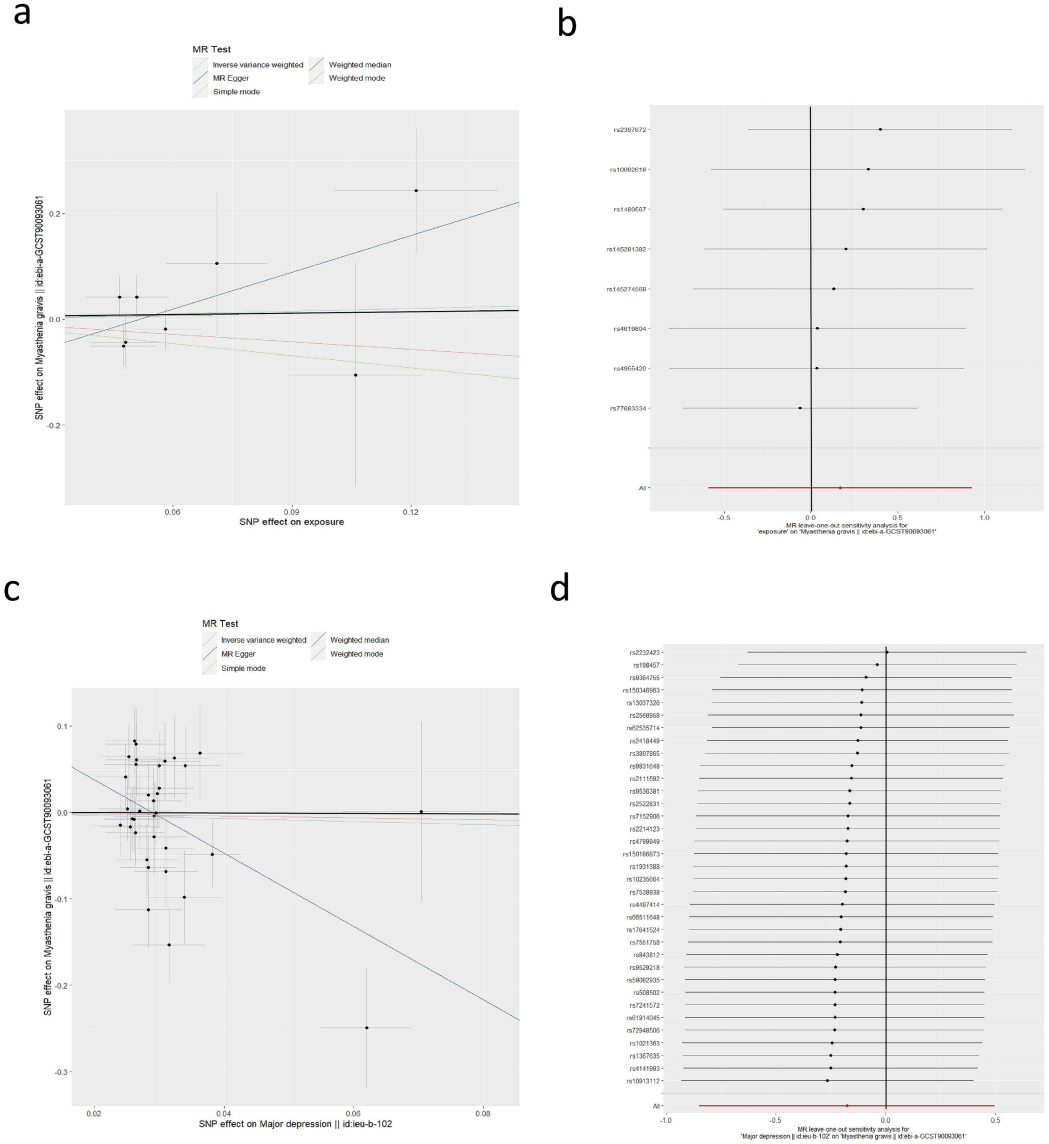
Causal effects of anxiety disorder and depression on MG. **(a)** Scatter plot of the causal relationship between anxiety disorder and MG using five MR methods. **(b)** Leave-one-out plot of the causal relationship between anxiety disorder and MG. **(c)** Scatter plot of the causal relationship between depression and MG using five MR methods. **(d)** Leave-one-out plot of the causal relationship between depression and MG.

For the pleiotropy detection of the effects between MG and anxiety disorder, no pleiotropy was observed (all *p* > 0.05) (Figures 3a,b). For pleiotropy detection of effects between MG and depression, the MR-Egger regression test was significant (*p* = 0.026), suggesting the possible presence of pleiotropy (Figure 3c). However, the MR-PRESSO analysis was not significant (*p* = 0.215). The LOO analysis did not identify any SNPs that significantly influenced the results (Figure 3d).

**Figure 4:**
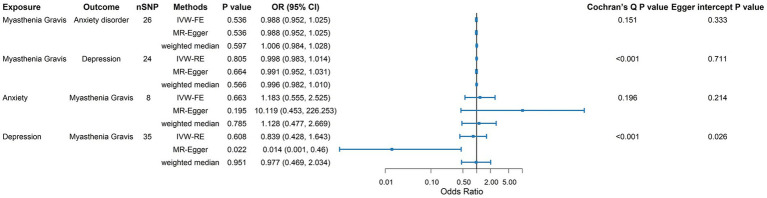
Combined forest plot summarizing the bidirectional MR analyses between MG and anxiety disorder/depression. For each exposure–outcome direction, causal effect estimates derived from inverse variance weighted (IVW), MR-Egger, and weighted median methods are presented as odds ratios (ORs) with 95% confidence intervals (CIs). Cochran’s *Q p*-values and MR-Egger intercept *p*-values are shown to indicate heterogeneity and potential horizontal pleiotropy, respectively. The vertical reference line indicates the null effect (OR = 1).

To provide an overall summary of the bidirectional MR results, we additionally present a combined forest plot summarizing the causal estimates from IVW, MR-Egger, and weighted median methods in each direction (Figure 4).

## 4 Discussion

The results of our real-world clinical study suggest that 62.5% of MG patients exhibited anxiety symptoms, while 46.90% presented depressive symptoms with MG; moreover, a meta-analysis including multiple studies reported a pooled prevalence of 36% for comorbid depression and 33% for comorbid anxiety, with individuals with MG tending to exhibit a high prevalence of depression and anxiety (4), which is consistent with our findings. Our results indicate that thymic abnormality status was not correlated with the presence of anxiety and depression. However, Li et al. reported that patients with MG and thymoma had a higher risk of anxiety and depression (23), which may be related to the fact that thymoma was included less in our study. Additionally, after adjusting for confounding variables, each 1-point increase in the QMG score was associated with a 11.9% higher odds of anxiety and a 15.7% higher odds of depression; each 1-point increase in the MG-ADL score was associated with a 22.5% higher odds of anxiety and a 18.5% higher odds of depression, indicating that poorer daily functioning and greater disease severity are correlated with anxiety and depression, in line with most prior studies (7, 24, 25). A plausible pathway is that greater MG severity may contribute to anxiety and depression indirectly through impairment of daily functioning; however, formal mediation analysis was not performed given the cross-sectional design, and this hypothesis should be tested in future longitudinal studies.

MR estimates the causal effect of lifelong genetic liability on disease risk and is not designed to capture the impact of disease severity, treatment exposure, disease duration, or psychosocial stress. Therefore, the absence of a genetic causal effect between MG and anxiety or depression should not be interpreted as evidence against the clinical associations observed in real-world studies. Rather, these findings indicate that such associations may arise from non-genetic mechanisms or pathways not captured by germline genetic instruments. Accordingly, clinical association should be distinguished from genetic causality, and the two lines of evidence are complementary rather than contradictory.

Research indicates that prolonged mental stress, anxiety, and depression can impede immune function via the HPA axis, sympathetic nervous system, and inflammatory pathways, potentially triggering MG (26, 27). Individuals with anxiety and depression may be at an elevated risk of developing MG compared with the general population. Currently, our clinical studies suggest associations between MG severity and anxiety and/or depression; however, whether a causal relationship exists between MG and anxiety and/or depression remains unknown. Accordingly, to further explore the potential causal relationship between the two, a MR analysis was conducted.

This study is the first to use bidirectional two-sample MR analysis to explore the causal relationships between genetically predicted risk of MG and anxiety disorder and depression. Our MR analysis did not find any statistically significant causal relationships between MG and anxiety disorder or depression. Various sensitivity and pleiotropy tests further confirmed the robustness of these results. Although we did not identify a positive causal link between these diseases, such efforts are crucial for understanding the complex associations between MG and psychiatric disorders. MR analysis, based on the random allocation of genetic variants during meiosis, can reflect lifelong exposure to risk factors, thereby minimizing the influence of reverse causality and confounding factors compared to observational studies. This provides more reliable evidence to clarify whether causal relationships exist (28). These findings raise the possibility that the observed comorbid associations in epidemiological studies may reflect non-causal associations driven by factors not captured by genetic liability, such as disease severity, treatment burden, or psychosocial stress, or by partial shared genetic architecture.

Glanville et al. (29) analyzed the genetic correlation between depression and 14 autoimmune diseases, including MG, using polygenic risk scores from the UK Biobank data. They found that the polygenic risk score for MG was significantly higher in patients with depression compared to the control group without depression. This suggests a genetic correlation between the prevalence of MG and depression, indicating a small shared genetic structure. Despite this, our MR analysis indicates no causal relationship between MG and the genetically predicted risk of depression.

When interpreting the results of previous epidemiological observations that reported higher rates of depression or anxiety in patients with MG, caution is warranted. Due to the fluctuating clinical symptoms and complications, patients with MG may experience psychological effects related to their adjustment and management of the condition. The complex treatments and invasive interventions during MG crises can impact their mental health. As the disease progresses, patients encounter more disabilities and issues in their treatment, which may reduce their quality of life and increase the incidence of psychiatric disorders such as depression and anxiety (4, 30).

Importantly, previous studies have suggested that MG and psychiatric disorders may share common pathogenic mechanisms. Inflammatory factors have long been considered significant pathological mediators in the development of MG (31, 32). They directly or indirectly initiate and activate immune cells, promoting the maturation, proliferation, and differentiation of specific B cells into plasma cells that produce specific antibodies, thereby leading to the occurrence of MG (33). Anti-interleukin-6 antibodies can reduce autoantibody levels and disease symptoms in experimental autoimmune MG rat models (34). On the other hand, chronic inflammation has been widely regarded as a potential driving factor for psychiatric disorders, with individuals diagnosed with psychiatric conditions frequently presenting with autoimmune diseases (35–37). There is a correlation between interleukin-6 levels and depression (38–41). This suggests that while MG and psychiatric disorders may share inflammatory pathways, further research is needed to clarify the nature of their association, as our MR analysis did not establish a direct causal link. These associations might be driven by similar pathogenic mechanisms.

This study has several limitations. Firstly, the clinical component was based on a relatively small, single-center, retrospective cohort with cross-sectional assessment of anxiety and depression at or near diagnosis. All enrolled patients had generalized MG and received low-dose corticosteroid therapy as standard baseline treatment, and key clinical covariates (e.g., immunosuppressant exposure, recent myasthenic crisis, or hospitalization history) were not uniformly available, precluding stratified or fully adjusted analyses, including by thymoma status. Secondly, the population data harnessed for this study derive exclusively from European GWAS, which can circumscribe the generalizability of these findings to diversified ethnic populations. Thirdly, while specific populations of MG patients possess antibodies for MuSK or LRP4, our study was confined to investigating patients exhibiting AchR antibodies. In addition, the currently available MG GWAS includes a relatively modest number of cases and aggregates heterogeneous serological subtypes, which may limit instrument strength and phenotype specificity in the MR analysis. Moreover, potential distinctions in genetic proportions could exist between various age or gender cohorts. Any disparities in proxy genotypes across different groups could potentially introduce biases into the MR analysis outcomes. Therefore, future endeavors should acquire larger and more comprehensive GWAS datasets that ensure adequate control for variables such as age and gender.

## 5 Conclusion

In our clinical study, patients with myasthenia gravis (MG) were more likely to exhibit anxiety and/or depression with increasing disease severity, and a significant association between the two was observed. However, based on our bidirectional Mendelian randomization (MR) analysis, no evidence of a causal relationship between MG and anxiety or depression was found in European-ancestry populations. Future studies should leverage larger-scale genome-wide association study (GWAS) resources and include non-European cohorts—particularly Chinese or broader Asian datasets—to assess associations in MG subgroups and related psychiatric disorders. These findings highlight the importance of distinguishing clinical association from genetic causality when interpreting psychiatric comorbidities in MG, and underscore that MR interrogates genetic liability rather than the lived experience, treatment burden, or psychosocial impact of MG. However, these conclusions should be interpreted with caution given the modest clinical sample size and the limited power and phenotype resolution of currently available MG GWAS datasets.

## Data Availability

The raw data supporting the conclusions of this article will be made available by the authors, without undue reservation.
