# Supplementary material for: Myasthenia gravis and anxiety-depression states: an integrated clinical and Mendelian randomization study
Source: Front Neurol. 2026 Mar 18;17:1791340. doi: 10.3389/fneur.2026.1791340 (PMC13038522; doi:10.3389/fneur.2026.1791340)
Supplement: Supplementary file 1 [file Table_1.doc]

| Table S1. STROBE-MR checklist.Item No.Section Checklist item Page No.Relevant text from manuscript1Title and AbstractIndicate Mendelian randomization (MR) as the study’s design in the title and/or the abstract if that is a main purpose of the study1The term "Mendelian randomization" was included both in the title and the abstract.INTRODUCTION2Background Explain the scientific background and rationale for the reported study. Is causality between exposure and outcome plausible? Justify why MR is a helpful method to address the study question.2Introduction, Paragraph 1-2.3Objectives State specific objectives clearly, including pre-specified causal hypotheses (if any). State that MR is a method that, under specific assumptions, intends to estimate causal effects2Introduction, Paragraph 3.METHODS4Study design and data sourcesPresent key elements of the study design early in the article. Consider including a table listing sources of data for all phases of the study. For each data source contributing to the analysis, describe the following:a) Setting: Describe the study design and the underlying population, if possible. Describe the setting, locations, and relevant dates, including periods of recruitment, exposure, follow-up, and data collection, when available.2Materials and methods, Section “Study design”. The flowchart of this study was also shown in Figure 1. Detailed information, such as periods of recruitment, follow-up, and data collection can be found in the original paper (Table 1).b) Participants: Give the eligibility criteria, and the sources and methods of selection of participants. Report the sample size, and whether any power or sample size calculations were carried out prior to the main analysis3Materials and methods, Section “Data sources”.c) Describe measurement, quality control and selection of genetic variants3Materials and methods, Section “Selection of instruments”.d) For each exposure, outcome, and other relevant variables, describe methods of assessment and diagnostic criteria for diseasesTable 1Detailed information, such as diagnostic criteria for diseases, can be found in the original paper (Table 1).e) Provide details of ethics committee approval and participant informed consent, if relevantTable 1No ethics approval was required for this study, as all data were obtained from published literature and publicly available summary data.5Assumptions Explicitly state the three core IV assumptions for the main analysis (relevance, independence and exclusion restriction) as well assumptions for any additional or sensitivity analysis-Figure 16Statistical methods: main analysisDescribe statistical methods and statistics useda) Describe how quantitative variables were handled in the analyses (i.e., scale, units, model)3Materials and methods, Section “Statistical analysis”b) Describe how genetic variants were handled in the analyses and, if applicable, how their weights were selected3Materials and methods, Section “Selection of instruments”c) Describe the MR estimator (e.g. two-stage least squares, Wald ratio) and related statistics. Detail the included covariates and, in case of two-sample MR, whether the same covariate set was used for adjustment in the two samples3Materials and methods, Section “Statistical analysis”d) Explain how missing data were addressed-Not applicable to our studye) If applicable, indicate how multiple testing was addressed-We did not perform correction of multiple comparisons.7Assessment of assumptionsDescribe any methods or prior knowledge used to assess the assumptions or justify their validity3Materials and methods, Section “Study design” and “Selection of instruments”8Sensitivity analyses and additional analysesDescribe any sensitivity analyses or additional analyses performed (e.g. comparison of effect estimates from different approaches, independent replication, bias analytic techniques, validation of instruments, simulations)3Materials and methods, Section “Statistical analysis”9Software and pre registrationa) Name statistical software and package(s), including version and settings used3Materials and methods, Section “Statistical analysis”b) State whether the study protocol and details were pre-registered (as well as when and where)-Not applicable to our studyRESULTS10Descriptive dataa) Report the numbers of individuals at each stage of included studies and reasons for exclusion. Consider use of a flow diagramFigure 1The flowchart of this study was also shown in Figure 1.b) Report summary statistics for phenotypic exposure(s), outcome(s), and other relevant variables (e.g. means, SDs, proportions)-Detailed summary statistics was also shown in Supplement file 1: Table S4c) If the data sources include meta-analyses of previous studies, provide the assessments of heterogeneity across these studies-Not applicable to our studyd) For two-sample MR:i. Provide justification of the similarity of the genetic variant-exposure associations between the exposure and outcome samples-These GWAS sample populations needed to be predominantly of European descent and largely independent of each otherii. Provide information on the number of individuals who overlap between the exposure and outcome studies-Not applicable to our study11Main resultsa) Report the associations between genetic variant and exposure, and between genetic variant and outcome, preferably on an interpretable scale4Results, Section “Selection of instrumental variables”. Summary statistics was also shown in Supplement file 1: Table S3b) Report MR estimates of the relationship between exposure and outcome, and the measures of uncertainty from the MR analysis, on an interpretable scale, such as odds ratio or relative risk per SD difference4 Results. Section "Causal effects of MG on anxiety disorder and depression" and "Causal effects of anxiety disorder and depression on MG".c) If relevant, consider translating estimates of relative risk into absolute risk for a meaningful time period-Not applicable to our studyd) Consider plots to visualize results (e.g. forest plot, scatterplot of associations between genetic variants and outcome versus between genetic variants and exposure)Figure 2 and Figure 3Scatterplots of genetic variants were shown in Figure 2 and Figure 3.12Assessment of assumptionsa) Report the assessment of the validity of the assumptions4Results, Section "Selection of instrumental variables". We also calculated the F statistic for each IV in Supplement file 1: Table S2.b) Report any additional statistics (e.g., assessments of heterogeneity across genetic variants, such as I2, Q statistic or E-value)4Results, Section "Causal effects of MG on anxiety disorder and depression" and "Causal effects of anxiety disorder and depression on MG". We also calculated the Q statistic in Supplement file 1: Table S3.13Sensitivity analyses and additional analysesa) Report any sensitivity analyses to assess the robustness of the main results to violations of the assumptions4, 5Results, Section "Causal relationship between MDD and CSVD" and "Causal relationship between CSVD and MDD". b) Report results from other sensitivity analyses or additional analyses-Supplement file 1: Table S3.c) Report any assessment of direction of causal relationship (e.g., bidirectional MR)4, 5Results, Section "Causal effects of MG on anxiety disorder and depression" and "Causal effects of anxiety disorder and depression on MG".d) When relevant, report and compare with estimates from non-MR analyses-Not applicable to our studye) Consider additional plots to visualize results (e.g., leave-one-out analyses)-Figure 2 and Figure 3.DISCUSSION14Key results Summarize key results with reference to study objectives5Discussion, Paragraph 115Limitations Discuss limitations of the study, taking into account the validity of the IV assumptions, other sources of potential bias, and imprecision. Discuss both direction and magnitude of any potential bias and any efforts to address them5, 6Discussion, Paragraph 2-416Interpretationa) Meaning: Give a cautious overall interpretation of results in the context of their limitations and in comparison with other studies5Discussion, Paragraph 1b) Mechanism: Discuss underlying biological mechanisms that could drive a potential causal relationship between the investigated exposure and the outcome, and whether the gene-environment equivalence assumption is reasonable. Use causal language carefully, clarifying that IV estimates may provide causal effects only under certain assumptions5Discussion, Paragraph 2-4c) Clinical relevance: Discuss whether the results have clinical or public policy relevance, and to what extent they inform effect sizes of possible interventions5Discussion, Paragraph 117Generalizability Discuss the generalizability of the study results (a) to other populations, (b) across other exposure periods/timings, and (c) across other levels of exposure6Discussion, Paragraph 5OTHER INFORMATION18Funding Describe sources of funding and the role of funders in the present study and, if applicable, sources of funding for the databases and original study or studies on which the present study is based6Section “Funding”.19Data and data sharingProvide the data used to perform all analyses or report where and how the data can be accessed, and reference these sources in the article. Provide the statistical code needed to reproduce the results in the article, or report whether the code is publicly accessible and if so, where6Section “Availability of data and materials”20Conflicts of InterestAll authors should declare all potential conflicts of interest6Section “Competing interests”This checklist is copyrighted by the Equator Network under the Creative Commons Attribution 3.0 Unported (CC BY 3.0) license.1. Skrivankova VW, Richmond RC, Woolf BAR, et al. Strengthening the Reporting of Observational Studies in Epidemiology Using Mendelian Randomization: The STROBE-MR Statement. JAMA. 2021;326(16):1614-1621. doi:10.1001/jama.2021.182362.Skrivankova VW, Richmond RC, Woolf BAR, et al. Strengthening the reporting of observational studies in epidemiology using mendelian randomisation (STROBE-MR): explanation and elaboration. BMJ. 2021;375:n2233. Published 2021 Oct 26. doi:10.1136/bmj.n2233 |
| --- |
|  |
|  |
|  |
|  |
|  |
|  |
|  |
|  |
|  |
|  |
|  |
|  |
|  |
|  |
|  |
|  |
|  |
|  |
|  |
|  |
|  |
|  |
|  |
|  |
|  |
|  |
|  |
|  |
|  |
|  |
|  |
|  |
|  |
|  |
|  |
|  |
|  |
|  |
|  |
|  |
|  |
|  |
|  |
|  |
|  |
|  |
|  |
|  |
|  |
|  |
|  |
|  |
|  |
|  |
|  |
|  |
|  |
|  |
|  |
|  |
|  |
|  |
|  |
