# Supplementary material for: Myasthenia gravis and anxiety-depression states: an integrated clinical and Mendelian randomization study
Source: Front Neurol. 2026 Mar 18;17:1791340. doi: 10.3389/fneur.2026.1791340 (PMC13038522; doi:10.3389/fneur.2026.1791340)
Supplement: Supplementary file 2 [file Table_2.docx]

| Table S2. Characteristics of the instrumental variables used in the bidirectional Mendelian randomization. | | | | | | | | | | | | | | |
| --- | --- | --- | --- | --- | --- | --- | --- | --- | --- | --- | --- | --- | --- | --- |
| Exposure | Outcome | nSNP | SNP | chr | pos | Exposure | | | | Outcome | | | | F-statistics |
|  |  |  |  |  |  | Effect Allele | Beta | SE | P value | Effect Allele | Beta | SE | P value |  |
| Myasthenia Gravis | Anxiety disorder | 26 | rs111263101 | 2 | 38313018 | G | 0.205 | 0.043 | 1.44E-06 | G | -0.005 | 0.011 | 6.28E-01 | 363.86 |
|  |  |  | rs112477645 | 4 | 10553688 | A | -0.293 | 0.059 | 7.40E-07 | A | -0.019 | 0.013 | 1.36E-01 | 606.04 |
|  |  |  | rs116976127 | 18 | 50360462 | T | 0.529 | 0.116 | 4.91E-06 | T | 0.028 | 0.020 | 1.64E-01 | 744.22 |
|  |  |  | rs11985475 | 8 | 25464656 | T | 0.172 | 0.038 | 4.23E-06 | T | -0.010 | 0.008 | 1.85E-01 | 565.16 |
|  |  |  | rs12637233 | 3 | 111438443 | A | 0.244 | 0.052 | 2.82E-06 | A | -0.004 | 0.014 | 7.88E-01 | 471.85 |
|  |  |  | rs12884697 | 14 | 61935996 | A | -0.263 | 0.053 | 6.23E-07 | A | -0.018 | 0.012 | 1.51E-01 | 614.02 |
|  |  |  | rs12901830 | 15 | 79239449 | G | 0.246 | 0.052 | 2.17E-06 | G | 0.009 | 0.009 | 2.92E-01 | 497.36 |
|  |  |  | rs138255937 | 9 | 92188847 | T | 0.557 | 0.121 | 3.85E-06 | T | 0.065 | 0.066 | 3.24E-01 | 475.64 |
|  |  |  | rs147362774 | 10 | 13560872 | C | 0.650 | 0.125 | 1.88E-07 | C | -0.018 | 0.037 | 6.27E-01 | 164.77 |
|  |  |  | rs17172212 | 7 | 36047213 | T | 0.662 | 0.143 | 3.44E-06 | T | 0.006 | 0.021 | 7.75E-01 | 337.97 |
|  |  |  | rs2476601 | 1 | 114377568 | G | -0.396 | 0.061 | 7.95E-11 | G | -0.005 | 0.011 | 6.20E-01 | 1272.71 |
|  |  |  | rs2523595 | 6 | 31326618 | A | -0.260 | 0.039 | 2.55E-11 | A | -0.001 | 0.008 | 9.34E-01 | 1236.95 |
|  |  |  | rs28597864 | 17 | 66801318 | C | 0.200 | 0.043 | 2.55E-06 | C | -0.011 | 0.009 | 2.21E-01 | 560.86 |
|  |  |  | rs35274388 | 2 | 175629220 | A | 0.450 | 0.081 | 3.07E-08 | A | 0.053 | 0.035 | 1.33E-01 | 459.50 |
|  |  |  | rs35695082 | 16 | 30792389 | C | 0.226 | 0.049 | 3.54E-06 | C | -0.010 | 0.010 | 3.15E-01 | 388.67 |
|  |  |  | rs3809717 | 17 | 37886986 | A | 0.203 | 0.039 | 2.63E-07 | A | 0.001 | 0.008 | 8.77E-01 | 684.57 |
|  |  |  | rs4377259 | 19 | 33981927 | C | 0.192 | 0.041 | 2.46E-06 | C | 0.013 | 0.008 | 9.47E-02 | 611.27 |
|  |  |  | rs4409785 | 11 | 95311422 | C | 0.255 | 0.045 | 1.54E-08 | C | 0.017 | 0.010 | 9.83E-02 | 611.03 |
|  |  |  | rs4574025 | 18 | 60009814 | T | -0.287 | 0.037 | 7.08E-15 | T | -0.006 | 0.008 | 4.54E-01 | 1633.14 |
|  |  |  | rs56074046 | 17 | 7358930 | A | 0.174 | 0.037 | 2.87E-06 | A | 0.002 | 0.008 | 8.11E-01 | 527.39 |
|  |  |  | rs6508442 | 18 | 24014065 | C | -0.171 | 0.037 | 4.62E-06 | C | -0.011 | 0.008 | 1.82E-01 | 496.12 |
|  |  |  | rs6590685 | 11 | 133169568 | A | -0.169 | 0.037 | 4.14E-06 | A | -0.011 | 0.008 | 1.50E-01 | 551.34 |
|  |  |  | rs76815088 | 6 | 32588713 | C | -0.866 | 0.113 | 1.58E-14 | C | 0.020 | 0.013 | 1.37E-01 | 3270.10 |
|  |  |  | rs7727530 | 5 | 8130353 | A | -0.330 | 0.068 | 1.04E-06 | A | 0.018 | 0.011 | 9.68E-02 | 840.09 |
|  |  |  | rs7869982 | 9 | 105474366 | A | 0.243 | 0.053 | 4.64E-06 | A | 0.001 | 0.013 | 9.22E-01 | 299.29 |
|  |  |  | rs912425 | 13 | 42997324 | A | 0.199 | 0.044 | 4.98E-06 | A | -0.014 | 0.010 | 1.60E-01 | 588.14 |
| Myasthenia Gravis | Major depressive disorder | 24 | rs112477645 | 4 | 10553688 | A | -0.293 | 0.059 | 7.40E-07 | A | -0.008 | 0.007 | 2.49E-01 | 606.04 |
|  |  |  | rs116976127 | 18 | 50360462 | T | 0.529 | 0.116 | 4.91E-06 | T | 0.018 | 0.014 | 1.82E-01 | 744.22 |
|  |  |  | rs12637233 | 3 | 111438443 | A | 0.244 | 0.052 | 2.82E-06 | A | -0.005 | 0.007 | 4.82E-01 | 471.85 |
|  |  |  | rs12884697 | 14 | 61935996 | A | -0.263 | 0.053 | 6.23E-07 | A | 0.012 | 0.007 | 7.43E-02 | 614.02 |
|  |  |  | rs12901830 | 15 | 79239449 | G | 0.246 | 0.052 | 2.17E-06 | G | 0.001 | 0.006 | 8.35E-01 | 497.36 |
|  |  |  | rs138255937 | 9 | 92188847 | T | 0.557 | 0.121 | 3.85E-06 | T | -0.006 | 0.018 | 7.47E-01 | 475.64 |
|  |  |  | rs147362774 | 10 | 13560872 | C | 0.650 | 0.125 | 1.88E-07 | C | 0.031 | 0.019 | 9.82E-02 | 164.77 |
|  |  |  | rs2245569 | 10 | 7452743 | G | 0.237 | 0.042 | 1.66E-08 | G | 0.014 | 0.005 | 7.77E-03 | 42.96 |
|  |  |  | rs2476601 | 1 | 114377568 | G | -0.396 | 0.061 | 7.95E-11 | G | 0.001 | 0.007 | 9.97E-01 | 1272.71 |
|  |  |  | rs2523595 | 6 | 31326618 | A | -0.260 | 0.039 | 2.55E-11 | A | 0.021 | 0.005 | 3.27E-06 | 1236.95 |
|  |  |  | rs28597864 | 17 | 66801318 | C | 0.200 | 0.043 | 2.55E-06 | C | 0.006 | 0.005 | 2.59E-01 | 560.86 |
|  |  |  | rs35274388 | 2 | 175629220 | A | 0.450 | 0.081 | 3.07E-08 | A | 0.002 | 0.012 | 8.85E-01 | 459.50 |
|  |  |  | rs35695082 | 16 | 30792389 | C | 0.226 | 0.049 | 3.54E-06 | C | -0.004 | 0.006 | 4.49E-01 | 388.67 |
|  |  |  | rs3809717 | 17 | 37886986 | A | 0.203 | 0.039 | 2.63E-07 | A | -0.007 | 0.005 | 1.65E-01 | 684.57 |
|  |  |  | rs4377259 | 19 | 33981927 | C | 0.192 | 0.041 | 2.46E-06 | C | -0.002 | 0.005 | 6.80E-01 | 611.27 |
|  |  |  | rs4409785 | 11 | 95311422 | C | 0.255 | 0.045 | 1.54E-08 | C | -0.004 | 0.006 | 4.74E-01 | 611.03 |
|  |  |  | rs4574025 | 18 | 60009814 | T | -0.287 | 0.037 | 7.08E-15 | T | -0.004 | 0.004 | 3.39E-01 | 1633.14 |
|  |  |  | rs56074046 | 17 | 7358930 | A | 0.174 | 0.037 | 2.87E-06 | A | 0.015 | 0.005 | 1.00E-03 | 527.39 |
|  |  |  | rs6508442 | 18 | 24014065 | C | -0.171 | 0.037 | 4.62E-06 | C | 0.004 | 0.005 | 3.43E-01 | 496.12 |
|  |  |  | rs6590685 | 11 | 133169568 | A | -0.169 | 0.037 | 4.14E-06 | A | -0.006 | 0.004 | 2.05E-01 | 551.34 |
|  |  |  | rs76815088 | 6 | 32588713 | C | -0.866 | 0.113 | 1.58E-14 | C | 0.006 | 0.010 | 5.25E-01 | 3270.10 |
|  |  |  | rs7727530 | 5 | 8130353 | A | -0.330 | 0.068 | 1.04E-06 | A | 0.008 | 0.008 | 3.03E-01 | 840.09 |
|  |  |  | rs7869982 | 9 | 105474366 | A | 0.243 | 0.053 | 4.64E-06 | A | -0.020 | 0.007 | 4.52E-03 | 299.29 |
|  |  |  | rs912425 | 13 | 42997324 | A | 0.199 | 0.044 | 4.98E-06 | A | 0.010 | 0.005 | 3.79E-02 | 588.14 |
| Anxiety disorder | Myasthenia Gravis | 8 | rs10092618 | 8 | 65583843 | G | 0.058 | 0.008 | 8.07E-12 | G | -0.019 | 0.038 | 6.20E-01 | 520.57 |
|  |  |  | rs145274568 | 2 | 197159986 | G | 0.071 | 0.013 | 2.69E-08 | G | 0.106 | 0.137 | 4.39E-01 | 69.16 |
|  |  |  | rs145281382 | 17 | 43044922 | C | 0.106 | 0.017 | 3.50E-10 | C | -0.105 | 0.212 | 6.19E-01 | 39.58 |
|  |  |  | rs1480567 | 16 | 73706848 | T | 0.048 | 0.009 | 2.46E-08 | T | -0.044 | 0.047 | 3.54E-01 | 266.71 |
|  |  |  | rs2397672 | 6 | 100341614 | G | 0.047 | 0.008 | 9.51E-09 | G | -0.051 | 0.039 | 1.91E-01 | 356.75 |
|  |  |  | rs4619804 | 3 | 18674644 | A | 0.051 | 0.008 | 3.37E-10 | A | 0.042 | 0.041 | 2.99E-01 | 350.66 |
|  |  |  | rs4955420 | 3 | 49208865 | T | -0.046 | 0.009 | 4.58E-08 | T | -0.042 | 0.038 | 2.77E-01 | 344.92 |
|  |  |  | rs77683334 | 12 | 23096287 | C | -0.121 | 0.021 | 5.06E-09 | C | -0.243 | 0.118 | 3.96E-02 | 299.70 |
| Major depressive disorder | Myasthenia Gravis | 35 | rs1021363 | 10 | 106610839 | G | -0.030 | 0.005 | 2.29E-11 | G | -0.054 | 0.039 | 1.62E-01 | 209.65 |
|  |  |  | rs10235664 | 7 | 2086814 | C | -0.027 | 0.005 | 4.68E-08 | C | -0.001 | 0.041 | 9.72E-01 | 152.61 |
|  |  |  | rs10913112 | 1 | 175913828 | T | -0.026 | 0.005 | 4.53E-09 | T | -0.083 | 0.038 | 3.01E-02 | 164.00 |
|  |  |  | rs13037326 | 20 | 44692598 | T | 0.031 | 0.005 | 2.40E-10 | T | -0.068 | 0.041 | 9.66E-02 | 168.91 |
|  |  |  | rs1367635 | 18 | 50861409 | C | 0.025 | 0.004 | 4.35E-09 | C | 0.064 | 0.037 | 7.84E-02 | 159.73 |
|  |  |  | rs150186873 | 6 | 27182377 | C | 0.070 | 0.012 | 4.51E-09 | C | 0.001 | 0.104 | 9.92E-01 | 49.58 |
|  |  |  | rs150346963 | 7 | 117625599 | T | 0.028 | 0.004 | 1.16E-10 | T | -0.064 | 0.038 | 8.84E-02 | 194.47 |
|  |  |  | rs17641524 | 1 | 197704717 | T | -0.030 | 0.005 | 1.50E-08 | T | -0.028 | 0.044 | 5.26E-01 | 133.96 |
|  |  |  | rs1931388 | 9 | 11203149 | G | -0.030 | 0.004 | 1.68E-11 | G | 0.001 | 0.038 | 9.79E-01 | 214.54 |
|  |  |  | rs198457 | 11 | 61471678 | T | -0.032 | 0.006 | 1.90E-08 | T | 0.154 | 0.044 | 4.76E-04 | 137.93 |
|  |  |  | rs2111592 | 2 | 208049581 | A | 0.026 | 0.005 | 1.35E-08 | A | -0.024 | 0.039 | 5.47E-01 | 140.39 |
|  |  |  | rs2214123 | 6 | 67000001 | G | -0.026 | 0.005 | 8.56E-09 | G | 0.008 | 0.038 | 8.39E-01 | 155.79 |
|  |  |  | rs2232423 | 6 | 28366151 | G | -0.062 | 0.007 | 1.14E-18 | G | 0.249 | 0.069 | 2.80E-04 | 333.95 |
|  |  |  | rs2418449 | 9 | 119731359 | C | -0.028 | 0.005 | 4.25E-09 | C | 0.055 | 0.041 | 1.78E-01 | 149.14 |
|  |  |  | rs2522831 | 7 | 82448100 | C | 0.024 | 0.004 | 2.11E-08 | C | -0.015 | 0.037 | 6.85E-01 | 142.63 |
|  |  |  | rs2568958 | 1 | 72765116 | A | 0.038 | 0.004 | 2.90E-18 | A | -0.049 | 0.039 | 2.07E-01 | 338.02 |
|  |  |  | rs3807865 | 7 | 12250402 | A | 0.031 | 0.004 | 1.09E-12 | A | -0.041 | 0.037 | 2.69E-01 | 237.49 |
|  |  |  | rs4141983 | 1 | 18122009 | C | -0.026 | 0.005 | 9.69E-09 | C | -0.079 | 0.041 | 5.47E-02 | 163.24 |
|  |  |  | rs4497414 | 11 | 88756779 | C | 0.029 | 0.004 | 2.93E-11 | C | 0.013 | 0.037 | 7.17E-01 | 210.12 |
|  |  |  | rs4799949 | 18 | 35155910 | T | -0.029 | 0.005 | 1.40E-10 | T | 0.004 | 0.039 | 9.14E-01 | 198.62 |
|  |  |  | rs508502 | 13 | 80921519 | T | -0.026 | 0.005 | 3.56E-08 | T | -0.055 | 0.041 | 1.71E-01 | 135.05 |
|  |  |  | rs59082935 | 7 | 38724868 | T | 0.036 | 0.007 | 3.07E-08 | T | 0.069 | 0.053 | 1.97E-01 | 160.09 |
|  |  |  | rs61914045 | 12 | 52352301 | A | 0.031 | 0.005 | 7.96E-09 | A | 0.059 | 0.045 | 1.88E-01 | 151.15 |
|  |  |  | rs62535714 | 9 | 37182655 | A | 0.034 | 0.006 | 4.69E-09 | A | -0.098 | 0.054 | 6.71E-02 | 155.81 |
|  |  |  | rs66511648 | 3 | 117515519 | C | 0.030 | 0.005 | 6.03E-10 | C | 0.022 | 0.041 | 5.95E-01 | 175.08 |
|  |  |  | rs7152906 | 14 | 75125540 | C | 0.026 | 0.004 | 1.87E-09 | C | -0.008 | 0.037 | 8.35E-01 | 156.74 |
|  |  |  | rs7241572 | 18 | 77580712 | A | 0.032 | 0.005 | 2.43E-09 | A | 0.063 | 0.047 | 1.85E-01 | 141.45 |
|  |  |  | rs72948506 | 2 | 212618440 | A | 0.027 | 0.005 | 1.71E-08 | A | 0.061 | 0.041 | 1.41E-01 | 149.81 |
|  |  |  | rs7538938 | 1 | 67132262 | C | 0.025 | 0.004 | 7.29E-09 | C | 0.004 | 0.037 | 9.16E-01 | 156.01 |
|  |  |  | rs7551758 | 1 | 52274078 | G | 0.028 | 0.004 | 5.11E-11 | G | 0.020 | 0.036 | 5.77E-01 | 197.90 |
|  |  |  | rs843812 | 3 | 61255413 | A | 0.025 | 0.004 | 1.41E-08 | A | 0.041 | 0.038 | 2.73E-01 | 135.72 |
|  |  |  | rs9364755 | 6 | 165117329 | G | 0.028 | 0.005 | 3.49E-08 | G | -0.113 | 0.045 | 1.22E-02 | 151.27 |
|  |  |  | rs9529218 | 13 | 31790053 | T | -0.034 | 0.005 | 2.23E-10 | T | -0.054 | 0.047 | 2.47E-01 | 172.08 |
|  |  |  | rs9536381 | 13 | 53860655 | T | 0.026 | 0.005 | 2.62E-08 | T | -0.017 | 0.040 | 6.73E-01 | 156.04 |
|  |  |  | rs9831648 | 3 | 49214303 | T | -0.029 | 0.005 | 1.59E-08 | T | 0.029 | 0.043 | 5.02E-01 | 161.05 |
