# Supplementary material for: Myasthenia gravis and anxiety-depression states: an integrated clinical and Mendelian randomization study
Source: Front Neurol. 2026 Mar 18;17:1791340. doi: 10.3389/fneur.2026.1791340 (PMC13038522; doi:10.3389/fneur.2026.1791340)
Supplement: Supplementary file 3 [file Table_3.doc]

**Table S3.** Univariable logistic regression analyses of factors associated with anxiety and depressive symptoms in patients with MG (binary outcomes)

| Variables | Anxiety | | | Depression | | |
| --- | --- | --- | --- | --- | --- | --- |
|  | B (coefficient) | OR (95% CI) | P value | B (coefficient) | OR (95% CI) | P value |
| age | 0.009 | 1.009（0.982-1.036） | 0.517 | 0.016 | 1.016  （0.990-1.043） | 0.238 |
| Sex | 0.318 | 1.375（0.593-3.186） | 0.458 | 0.223 | 1.250（0.557-2.806） | 0.589 |
| Disease duration | 0.005 | 1.005（0.953-1.060） | 0.854 | 0.005 | 1.005（0.955-1.058） | 0.847 |
| MGFA classifications | 0.605 | 1.831（0.793-4.226） | 0.157 | 0.682 | 1.977（0.876-4.463） | 0.101 |
| With or without thymic abnormalities | 0.128 | 1.136（0.467-2.765） | 0.778 | 0.090 | 1.094（0.464-2.576） | 0.838 |
| Corticosteroid use (yes/no) | -0.113 | 0.893（0.388-2.056） | 0.790 | -0.209 | 0.812（0.360-1.829） | 0.614 |
| QMG scores | 0.120 | 1.128（1.032-1.232） | 0.008 | 0.155 | 1.168（1.064-1.283） | 0.001 |
| MG-ADL scores | 0.214 | 1.239（1.064-1.442） | 0.006 | 0.187 | 1.205（1.042-1.394） | 0.012 |

**Notes:** Anxiety was coded as 1 = anxiety (HAMA ≥ 14) and 0 = non-anxiety (HAMA < 14). Depression was coded as 1 = depression (HAMD ≥ 20) and 0 = non-depression (HAMD < 20). OR, odds ratio; CI, confidence interval.
