# Supplementary material for: Myasthenia gravis and anxiety-depression states: an integrated clinical and Mendelian randomization study
Source: Front Neurol. 2026 Mar 18;17:1791340. doi: 10.3389/fneur.2026.1791340 (PMC13038522; doi:10.3389/fneur.2026.1791340)
Supplement: Supplementary file 4 [file Table_4.doc]

| Table S4. Results of the pleiotropy and heterogeneity test results for myasthenia gravis and mood disorders. | | | | | | | | | |
| --- | --- | --- | --- | --- | --- | --- | --- | --- | --- |
| Exposure | Outcome | | nSNP | | Cochrane's Q | | MR Egger regression | | MR PRESSO Global test |
|  |  |  |  |  | Q value | P value | intercept | P value | P value |
| Myasthenia Gravis | | Anxiety disorder | | 26 | 32.238 | 0.151 | 0.005 | 0.333 | 0.139 |
| Myasthenia Gravis | | Major depressive disorder | | 24 | 70.560 | ＜0.001 | 0.002 | 0.711 | 0.095 |
| Anxiety disorder | | Myasthenia Gravis | | 8 | 9.875 | 0.196 | -0.119 | 0.214 | 0.401 |
| Major depressive disorder | | Myasthenia Gravis | | 35 | 72.349 | ＜0.001 | 0.122 | 0.026 | 0.216 |
| Abbreviations: nSNP, number of SNP. | | | | | | | | | |
